# Sequential Gastroenteritis Episodes Caused by 2 Norovirus Genotypes

**DOI:** 10.3201/eid2006.131627

**Published:** 2014-06

**Authors:** Gabriel I. Parra, Kim Y. Green

**Affiliations:** National Institutes of Health, Bethesda, Maryland, USA

**Keywords:** Norovirus, diarrhea, immunity, genotype, viruses

## Abstract

We investigated sequential episodes of acute norovirus gastroenteritis in a young child within an 11-month period. The infections were caused by 2 distinct genotypes (GII.4 and GII.6). Failure to achieve cross-protective immunity was linked to absence of an enduring and cross-reactive mucosal immune response, a critical consideration for vaccine design.

Noroviruses are major pathogens associated with acute gastroenteritis in persons of all ages. It is estimated that each year in developing countries, noroviruses are responsible for up to 200,000 deaths of children <5 years of age (*1*). Moreover, in the United States, because of the successful implementation of vaccination against rotaviruses, noroviruses have emerged as the leading cause of severe gastroenteritis requiring medical intervention among infants and young children (*2*).

Noroviruses are genetically diverse, and differences in the major capsid protein (VP1) have led to their classification into 6 genogroups (GI–GVI) and ≈30 genotypes. Noroviruses from genogroups GI, GII, and GIV infect humans; worldwide, GII.4 is the most prevalent genotype (*3*–*5*). Expression of VP1 results in self-assembly of virus-like particles that have been used to examine structural and antigenic differences among genotypes (*3*,*6*–*8*). However, lack of an in vitro cell culture system has hindered the ability to establish serotype differences by neutralization. Initial evidence for the existence of at least 2 distinct norovirus serotypes came from early studies among volunteers; these studies showed that infection with Norwalk or Hawaii viruses (representing GI and GII, respectively) did not induce cross-protection (*9*). Evidence also exists for the periodic emergence of new GII.4 strain variants that cause large global epidemics, possibly driven by escape from herd immunity (*5*,*10*). Further understanding of the natural history of these viruses is needed to establish the potential role of genotypic and antigenic variation in vaccine development.

## The Study

On January 15, 2012, a 13-month-old girl enrolled in a childcare center in Rockville, Maryland, experienced 3 episodes of vomiting within 1 hour, after which she had diarrhea or loose stools for ≈1 week. Within 24 hours after this child’s onset of symptoms, 2 family members reported multiple episodes of vomiting and diarrhea that lasted >3 days. Because several children and teachers at the childcare center reported similar symptoms, parents of children enrolled at the childcare center were alerted to the possibility of a gastroenteritis outbreak.

**Figure 1:**
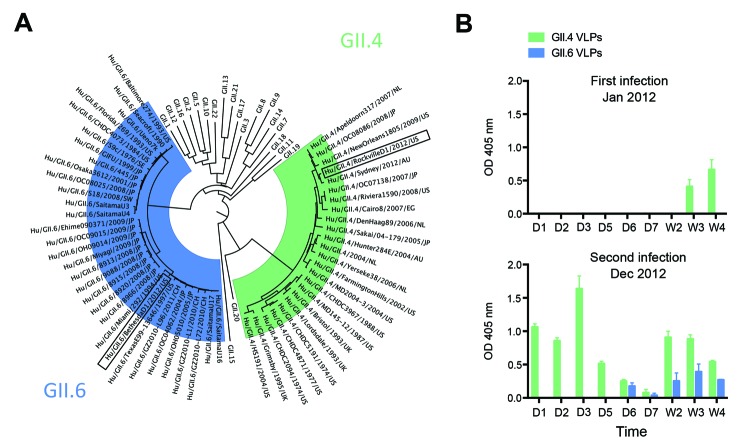
Characterization of norovirus detected in stool samples and levels of local IgA responses for each infection. A) Phylogenetic tree of the major capsid protein (VP1) region from representative norovirus strains from each of the 22 genotypes within strain GII. Representative strains from each GII.4 and GII.6 cluster were compared with the strains reported in this article (boxed). For each strain, the name/year/country of isolation are shown. B) Levels of IgA in feces collected during the first (G11.4) and (G11.6) second infections. ELISA plates were coated with 1 μg/mL of each virus-like particle (VLP). Fecal samples were collected daily (D) and weekly (W), diluted to 1:500 in phosphate-buffered saline (pH 7.4), and tested for the presence of IgA with a polyclonal anti-human IgA conjugate. The experiment was performed twice in duplicate wells. Bars represent mean; error bars represent the standard errors of the mean. OD, optical density.

The patient reported here was subsequently enrolled in National Institutes of Health clinical study NCT01306084, after receipt of informed consent from the mother. Fecal samples were collected from the child and examined for norovirus RNA by reverse transcription PCR. Viral RNA was detected for 4 weeks after the onset of symptoms, and viral RNA quantification reached up to 1.7 × 10^8^ genome copies/g feces. Sequence and phylogenetic analyses of VP1 from the virus (designated norovirus Hu/GII.4/RockvilleD1/2012/U.S.) showed that it grouped within the newly emerging virus GII.4 Sydney_2012 cluster (Figure 1, panel A) (*11*).

On December 10, 2012, a gastroenteritis outbreak occurred in a different childcare center in Bethesda, Maryland, at which the same child (now 24 months of age) was enrolled. The child experienced vomiting, diarrhea, and fatigue that lasted ≈2 days, and a similar disease pattern developed in a family member 24 hours after the onset of the child’s symptoms. Fecal samples were again positive for norovirus with 5.3 × 10^9^ genome copies/g of feces, and viral RNA was detected up to 3 weeks after disease onset. Phylogenetic and sequence analyses revealed a GII.6 norovirus (designated norovirus Hu/GII.6/BethesdaD1/2012/U.S.), most closely related to GII.6 noroviruses detected in Miami (Florida, USA) and Texas (USA) in 1994 and 1997, respectively (Figure 1, panel A).

**Figure 2,:**
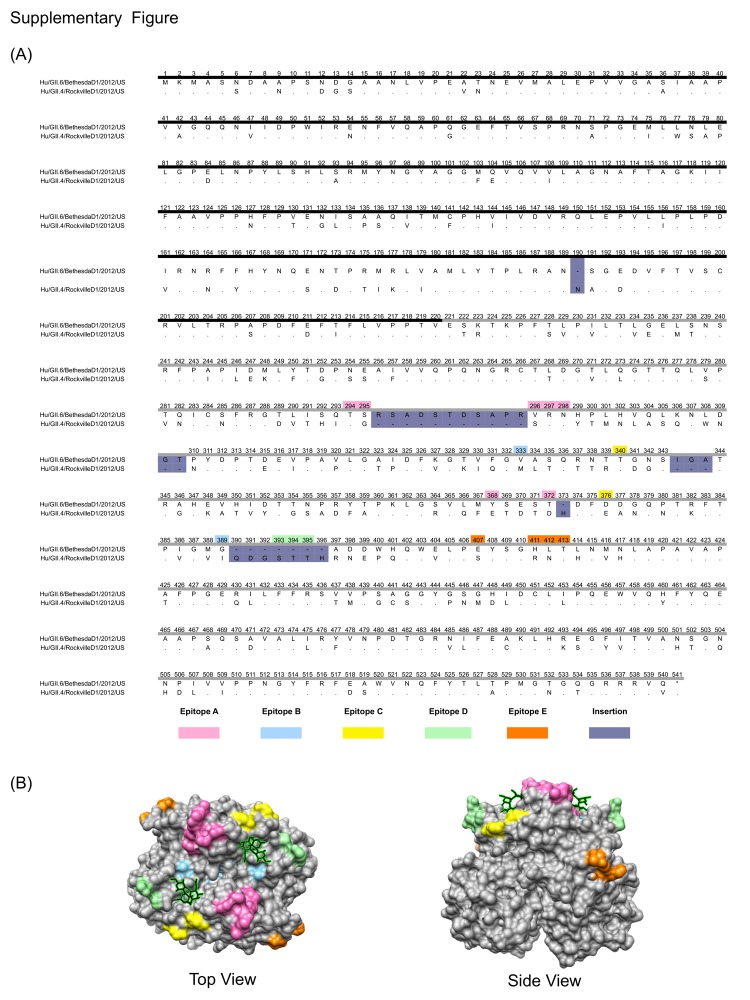
Appendix. Differences in the major capsid protein (VP1) between norovirus strains GII.4 and GII.6. A) Amino acid sequence alignment of the VP1 sequences. The shell (S) domain is highlighted with a dark line, and the protruding (P) domain is highlighted with a gray line. The color code for each of the epitopes and insertions is indicated. Residue numbers are based on norovirus strain GII.4. B) Top and side views of the P domain of GII.4 norovirus showing the location of the epitopes and the carbohydrate (represented as sticks and highlighted in green) binding sites.

Norovirus strains GII.4 and GII.6 differed by ≈38% in VP1 sequences; most amino acid sequence variation occurred in the capsid protruding domain (29%; 163/556). Alignment of the VP1 sequences from the 2 strains in this study showed several gaps; each strain bore discrete regions of amino acid insertions or deletions that differed from those in the other strain. The GII.6 VP1 (547 aa long) contained 3 insertions at positions 296 (11 residues), 310 (2 residues), and 344 (3 residues); GII.4 VP1 (540 aa long) did not contain these insertions. The same alignment showed GII.4 VP1 insertions at positions 190 (1 residue), 373 (1 residue), and 390 (7 residues) (Figure 2, Appendix). Of note, most gaps in the alignment of the VP1 sequences were present in or near recently described GII.4 epitopes (*7*,*12*), thereby suggesting that these residues might play a role in defining the antigenic specificity of the 2 genotypes.

To understand the absence of immunity to the second norovirus and to gain insight into the specificity of the mucosal immune response after sequential norovirus infections, we developed virus-like particles from both noroviruses. We used the Bac-to-Bac Baculovirus Expression System (Invitrogen, Carlsbad, CA, USA) and baculovirus–infected Sf-9 cells for virus-like particle production as described elsewhere (*7*). The corresponding virus-like particles were used to test for IgA in feces with an ELISA that used polyclonal goat anti-human IgA conjugated with horseradish peroxidase (1:2,000 dilution; KPL, Gaithersburg, MD, USA) as the detector antibody and 2,2′-azino-bis(3-ethylbenzthiazoline-6-sulfonic acid) (ABTS; KPL) as the substrate. During the child’s first infection with norovirus GII.4, virus-specific IgA was not detectable until 3 weeks after infection (Figure 1, panel B). At the onset of symptoms during the second infection with norovirus GII.6, a rapid anamnestic response specific for GII.4 virus-like particles developed. The GII.6-specific IgA response was detected at day 6 after infection, and IgA was detectable in feces until the last daily collection at 4 weeks (Figure 1, panel B). Examination of feces for norovirus-specific IgG after the second infection showed a similar anamnestic response that endured throughout the period examined (data not shown).

The child’s clinical history, young age, and absence of early detectable norovirus-specific fecal IgA suggest that she experienced a primary infection with GII.4 norovirus in the first childcare center outbreak. During the second infection with GII.6 norovirus, a rapid anamnestic GII.4-specific IgA response developed and persisted up to 4 weeks as the slower primary GII.6-specific IgA response developed ≈1 week after symptom onset. Follow-up fecal samples collected at 14 weeks after the second infection contained little or no detectable levels of either GII.6- or GII.4-specific IgA, indicating an eventual decline in mucosal IgA titers against each genotype (data not shown).

Previous data from human volunteer studies suggest that homologous immunity to Norwalk virus (genotype GI.1) lasts from 2 months to 2 years (*13*) and that a rapid and specific mucosal IgA response (likely anamnestic) was a correlate of protection (*14*). The data presented here are consistent with the development of a genotype-specific, short-lived mucosal IgA response to norovirus infection that, when stimulated anamnestically, might provide little or no protection against other norovirus genotypes.

## Conclusions

This study shows that a young child can experience 2 episodes of acute gastroenteritis caused by distinct norovirus genotypes (GII.4 and GII.6) within 1 year. Reinfection with distinct genotypes can commonly occur in younger persons, as recently demonstrated in a longitudinal study of norovirus infection in infants and young children in Peru (*15*). Genotypes within the 2 major genogroups of human noroviruses might represent distinct serotypes. The mechanisms of enduring norovirus immunity in the development of cross-protective and effective vaccines need to be elucidated.
